# Triangular Trajectory of Sustainable Development: Panel Analysis of the OECD Countries

**DOI:** 10.3390/ijerph18052374

**Published:** 2021-03-01

**Authors:** Taewook Huh, Yun Young Kim

**Affiliations:** 1 Department of Public Administration, Gyeongsang National University, Jinju City 52828, Korea; twhuh@gnu.ac.kr; 2 Department of Social Welfare, Jeonbuk National University, Jeonju City 54896, Korea

**Keywords:** sustainable development, ecological modernization, eco-socialism, panel analysis, OECD countries

## Abstract

This study analyzes how the three pillars of sustainable development (economic growth, social justice, and environmental protection) have influenced each other for the past twenty-six years (from 1987 to 2013). The relationship between the triangular pillar of SD can be characterized by “ecological modernization”, “eco-socialism”, and the traditional debate between growth and distribution. This paper examined the correlation analysis of the nine representative variables in the three categories, adopting the cases of twenty-six OECD countries. In particular, the panel analysis (PCSE models) was conducted to identify the seven independent determinants affecting both response (dependent) variables and environmental factors (“CO_2_ emissions” and “renewable electricity output”). In short, during the entire period, the findings reveal that all economic and social variables did not have a positive impact on reducing CO_2_ emissions. However, the variables of “employment in industry” and “social expenditure” are effected by the increase of renewable electricity output. Consequently, highlighting the detailed findings different for each set period (1987–2013, 1987–2002, and 2003–2013), this study suggests the implications of the analysis result in the light of the theories of ecological modernization and eco-socialism.

## 1. Introduction

At present, it is widely believed that until the middle of 20th century, we were required only to tackle problems of growth, because of the dominant trend of economic growth, and that during the late 20th century, we were required to deal with both growth and distribution problems. However, in the 21st century, a new dimensional problem-solving approach is needed in order to handle environmental problems as well as growth and distributional ones. Since the 1990s, in the context of globalization, countries have paid greater attention to sustainable development (hereafter SD), seeking for ecological and democratic development [1,2,3]. Today, the global development paradigm may be in a period of transition to ecological and grass-roots development [1,3]. 

The concept of sustainable development was first introduced through “Our Common Future” published by the World Commission on Environment and Development (WCED) in 1987 [4]. Sustainable development is now used as a universal value and provides the direction for human society to pursue the balance of economy, society, and environment. Thereafter, in June 1992, the United Nations Conference on Environment and Development (UNCED) was held in Rio, Brazil, where sustainable development was discussed in earnest. Ten years later, in August 2002, the World Summit on Sustainable Development (WSSD) was held in Johannesburg, South Africa, and the Johannesburg Declaration (Political Declaration) and implementation plan were adopted [5].

As the SD discussions expanded and developed, the international community became concerned not only with developing countries, but also with development goals that could comprehensively discuss and contain the sustainability of all humanity. Eventually, after a lot of discussion, the UN General Assembly in 2015 agreed on the global development system since 2015 (when Millennium Development Goals from 2000 end), and derived Sustainable Development Goals (hereafter SDGs) with 17 new goals and 169 targets [6].

Sustainable Development (SD), therefore, not only aims to prevent environmental deterioration and social conflict, but also provides a future-oriented development strategy. It requires a longer-term perspective with regards to the consequences of today’s actions, and that it provides a blueprint for harmoniously achieving economic growth, social integration, and environmental conservation [4]. As stated in the WCED in 1987, SD addresses various needs, for example, the essential need for economic growth in certain places, the human need to allow societies to provide equitable opportunities for all, and the basic need of those striving for a better quality of life [4]. Since 1987, the constant process of redefinition and interpretation has been carried out by exploring the interplay between different sub-principles of SD: “alleviating chronic poverty”, “encouraging public participation in decision making”, “observing important environmental limits to growth”, and “integrating an environmental dimension into all sectoral policy making” [7].

Looking at the development process of SD, this paper explores the following research questions.

- How has the relationship among the three pillars of SD (economic–social–environment sectors) in the OECD countries changed since SD officially appeared in the World Conference on Environment and Development (WCED) in 1987?
- Did the economic sector positively affect the environment sector (particularly climate change) in time-series?
- Did the social sector positively affect the environment (particularly climate change) in time-series?

This study delivers the findings of the correlation and causal relationship between the determinants by utilizing the panel analysis. Both the PCSE (panel corrected standard errors) models and STATA (software for statistics and data science) version 16.0 (Texas, USA) were used. Twenty-six OECD countries (valid data out of all thirty-seven OECD member countries) were included as cases by extracting the relevant data (nine variables: three variables of the economic sector, four variables of the social sector, and two variables of the environment sector (“CO_2_ emissions” and “renewable electricity output”)) on the three categories of sustainable development (SD). 

This paper focuses on the reduction of greenhouse gas (GHG) to cope with climate change, which has a wide range of effects on the global environment and ecosystem. As a representative global effort to GHG emissions, the OECD member countries were listed in the Annex II (twenty-four) countries or Annex I (forty-two) countries (with the preemptive GHG reduction obligations) to the Convention (UNFCCC: UN Framework Convention on Climate Change) adopted at the 1992 UNCED (UN Conference on Environment and Development). After the Kyoto Protocol of UNFCCC in 1997, the OECD member countries have been actively making efforts to reduce GHG (including CO_2_) emissions that are directly affecting climate change. The international community’s recent efforts to address climate change have come to fruition, as the Paris Agreement was adopted at the 21st Conference of the Parties to UNFCCC in 2015, and this provided a turning point for a coordinated global response to climate change [8,9]. In addition, this study underlines that the energy sector is responsible for more than 2/3 of GHG in the world, and has been drawing attention as a necessary sector to consider [10,11]. In other words, an expansion of renewable energy is required by reshuffling the existing fossil fuel-oriented energy system [12,13]. 

This study is composed as follows: in Section 2, it explains the theoretical background and issues of the perspectives of Sustainable Development (SD), the three pillars of SD, and eco-socialism and ecological modernization. Sustainable development has been emphasized only in the context of the theory of ecological modernization in the previous studies. However, this paper also introduces the theory of ecological socialism and encompasses multiple perspectives related to SD. In Section 3, it describes the application of the panel analysis method and sets up the measurement framework including the nine detailed variables in the three categories. In Section 4, this paper presents the research findings of the PCSE models including the entire period (1987–2013), and also the first (1987–2002) and second periods (2003–2013), and consequently the conclusions and implications in Section 5.

## 2. Theoretical Background and Issues 

### 2.1. The History and Notions of Sustainable Development 

The term “sustainable development” first appeared in the report, Our Common Future (known as the Brundtland Report), which was published by the World Conference on Environment and Development (WCED) in 1987. In terms of the evolution of the concept, as Mebratu [14] has explained, the notion of SD can be divided into three historical periods: “Pre-Stockholm (pre-1972)”, “from Stockholm to the World WCED (1972–1987)”, and “Post-WCED (1987–present)”. The first period refers to the time before the 1972 UN Conference on Human Environment (UNCHE) in Stockholm. In the second period, from Stockholm to the WCED, the UNCHE (or Stockholm Conference) used the slogan “Only One Earth”, and this became a catalyst in developing the concept of SD, wherein the Club of Rome was constituted and produced “The Limits to Growth”, a comprehensive report on the state of the natural environment. A few years later, in 1987, the term “sustainable development” was officially announced in a report by the WCED, “Our Common Future”. This systematically defined SD as “development that meets the needs of the present without compromising the ability of future generations to meet their own needs” [4], and this definition has been in common use throughout the world ever since. 

After the turning point of the WCED, active, global discussion about SD increased, and another ground-breaking event, the UN Conference on Environment and Development (UNCED), was held in Rio de Janeiro in 1992, known as the Rio Conference or the Earth Summit. The Rio Conference made SD its main agenda and produced two conventions (the Framework Convention on Climate Change and the Convention on Biological Diversity) and three non-binding instruments (the Declaration on Environment and Development (Rio Declaration), Agenda 21, and the Statement of Forest Principles) [5,15]. As a result, the term “Environmentally Sound and Sustainable Development” (ESSD) became used around the world. A decade after the Rio conference, the World Summit of Sustainable Development (WSSD) took place in Johannesburg in 2002, assessing the implementation performance over the preceding decade and formulating future implementation plans [16]. Additionally, in 2012, Rio+20 (UN Conference on SD) took place in Rio de Janeiro, Brazil, which launcehd a process to develop a set of Sustainable Development Goals (SDGs).

Thereafter, in 2015, nations around the world undertook another bold challenge. At the 70th UN General Assembly, 17 Sustainable Development Goals (SDGs) and 169 targets were defined as the common objective for the global community suffering from polarization, social conflict, and climate change. Every nation worked actively and profusely to achieve this goal. 

The UN’s Post-2015 Development Agenda is a new development goal to be pursued by the international community over the next 15 years by 2030 on behalf of the Millennium Development Goals (MDGs), which ended in 2015. The SDGs are a part of the Post-2015 Development agenda. The SDGs emphasize that sustainable development should be mainstreaming throughout the UN system and become a driver for its implementation, while focusing on priority areas to bring about sustainable development [6].

The UN SDGs include comprehensive and comprehensive planning goals. For this reason, they are also criticized for listing the proposed plans. Each country’s political, social, cultural, and historical backgrounds are different, and it is difficult to guarantee the will for action for economic reasons [17,18]. Therefore, international consensus and agreements have followed to achieve this goal; for example, the World Food and Security Commission, the Rome Declaration on Nutrition and the Framework for Action (2014. Nov.), the Conference of the Parties to the Climate Change Convention (UNFCCC), the 21st UNFCCC Paris Convention (2015. Dec.), UN Habitat, and the 2016–2036 New City Agenda (2016. Oct) [17].

Although the idea of SD has been globally embraced and used by a number of institutions since the Brundtland Report in 1987, there has been much dispute about the meaning of the concept. As [19] have explained, the term “development” is often recognized as a synonym of economic growth, and therefore SD can be regarded as ameliorating but not challenging economic growth. In this sense, the notion of SD in itself has a contradictory nature [15].

### 2.2. Three Pillars of Sustainable Development and Eco-Socialism and Ecological Modernization

Sustainable development (SD) has significant, integrated policy objectives that bring diverse interests together, and it regards environmental, social, and economic development as a single issue [1,4,14]. Sustainable development is not only an environmental issue, but one which embraces all types of policies from those based on environmental and social concerns as well as economic ones. As a result, as [16] have stated, the requirements of sustainability are multiple and interconnected, the pursuit of sustainability hinges on integration, and the core requirements and general rules must be accompanied by context specific elaborations. This conception of SD ultimately states that the existing economic growth-centered paradigm should be transformed into a new paradigm in which the economy and society develop together.

In order to succeed, sustainability requires a complete change in the dimensions of the social system. As social ecologists like [20] have argued, an environmental problem is also a social problem, because environmental problems will not cease to exist without first changing the hierarchy in which human beings rule over nature, a hierarchy which is, in turn, derived from a structure in which human beings try to rule other human beings. Hence, to tackle environmental problems, we must alter the social system, and society itself, rather than scrutinizing them. Following this train of thought, certain environmental problems must first be dealt with in relation to three dimensions of sustainability: ecological, economic, and social sustainability [14,21,22]. Social sustainability is subject to the dynamics of change, the diversity of actors interests, and relies on an efficient and responsible capacity for consensus building and implementation [21]. The capacity for social sustainability can be brought about by creating institutions that establish a framework and rules for social activities and deliver value [23,24]. In this regard, as White [25] has stated, institutions not only provide opportunities for changing individuals and society, but are also the motivating mechanism for bringing about these changes.

It is important to emphasize that putting SD into practice is not a simple process, because the concept deals with three interdependent pillars at the same time: economic growth, environmental conservation, and social development. Trade-offs between environmental, economic, and social policies are not easily made, therefore making it difficult for governments struggling to take explicit methodological approaches to introduce SD policies. As [26] have argued, applying the circularity and richness of sustainability into practical, linear projects often results in something being lost in the compromise. This is shown to bring about an inevitable conflict between sustainability and accountability, which are rooted in different value systems.

**Figure 1:**
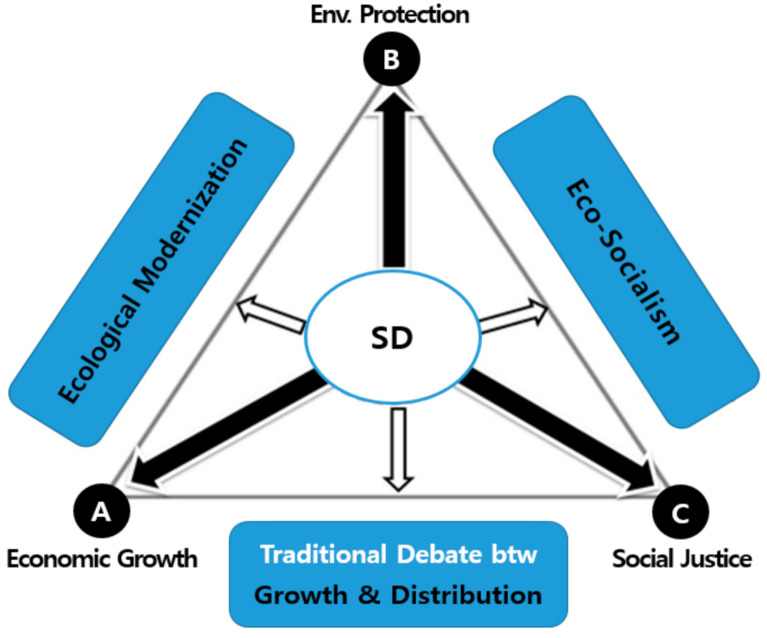
Three pillars of sustainable development and theoretical issues.

The concept of SD has been used in various ways, which can be interpreted as in Figure 1 below, depending on which of the three dimensions/pillars (economic growth, environmental protection, social justice) of SD is emphasized [26]. Sustainable development can be regarded as a paradigm or strategy to achieve the three goals in a balanced manner, while the area of SD (center circle) encompasses the direction and characteristics of the three sides of the triangle (Figure 1 below).

The side AB of the triangle of SD is characterized by “ecological modernization”, which recognizes the environmental hazards produced by economic activities and suggests a transition to both preventive political and social systems. This promotes the view that economic growth and environmental protection can be integrated into industrial modernity and that both can evolve into a win–win relationship [27,28,29]. In addition, the side BC of the triangle of SD has the meaning of eco-socialism, which seeks for the legitimacy of human protection from environmental risks, and tackling inequality to simultaneously realize social justice and environmental conservation [27,28,30]. Additionally, it can be seen that the side AC of the triangle represents the debate between traditional growth and distribution [27].

This paper underlines the contexts of ecological modernization and eco-socialism. First, the theory of ecological modernization is essentially in line with technology-orientation and deepens ecological rationalization in capitalism [28,30]. It also emphasizes the rational utilization and management of the environment [31,32]. Ecological modernization, a concept first proposed by Joseph Huber [33,34], originated from the perception that a sustainable society cannot be achieved by conducting traditional environmental policies alone. The theory of ecological modernization aims to ultimately overcome the inevitable reality of environmental destruction in the modern industrial society through institutional transformation and seeks technical solutions with more effective institutions to manage (control) the environment [35]. This is the greening of corporate ethics and goals and in line with a reformist position that believes in the self-correcting potential of capitalist modernization [34,35].

In particular, ecological modernists believe that ecological crisis can be overcome by technological and procedural innovation. Emphasis is placed on ecological efficiency, a strong incentive to utilize innovation that can generate more wealth while using less resources. The theory of ecological modernization has been established as a framework for major theories and a policy analysis of environmental policy [35]. It is significant that the ecological modernization theory can identify the social and structural characteristics of environmental problems and integrate them to pursue a harmonious relationship between environmental conservation and economic growth [31,34,35]. Furthermore, the new perceptions of “sustainable development”, “ecological efficiency” (efficiency of resource use, minimization of waste and pollutant emissions), and the roles of new actors (NGO activities such as environmental civic groups and residents’ autonomous organizations) have formed from the 1992 Earth Summit—United Nations Conference on Environment and Development, held in Rio de Janeiro, Brazil. Through this, the theory of ecological modernization has attracted attention as an important theoretical resource for analyzing and evaluating environmental policies [27,34].

Second, the theory of eco-socialism can be regarded as one of the most representative theories based on the logic of ecologicalism or environmentalism that requires the fundamental transformation of the political, economic, and social systems [36]. Eco-socialism emphasizes the aspect of combining “green” thinking with “red” thinking. Fundamentally, the value of the ecological orientation is anti-capitalist. It leads to the environmentalists’ outlook on the future that forms a common basis within the socialist tradition of decentralization, communism, and leftist socialism [28,36].

Eco-socialism is particularly the main idea of the left-wing green party and environmental activists, and eco-socialists advocate the preservation and the ecological preservation of the capitalism of indiscriminate industrialization that leads to an ecological crisis [37,38]. Eco-socialism includes alternative globalization, values economic development and harmony of ecology, and advocates liberal and socialist policies [36]. Eco-socialism is also called red greens [27,39]. Like the purpose of green politics, social justice is valued, and it is argued that rapid development to pursue unconditional profits of capitalism destroys the environment beyond rapid development [36,37]. According to the eco-socialists, it is necessary to pursue environmental development at the same time as development speed suitable for human minimum utility [36,39].

## 3. Methodology and Measurement Framework

### 3.1. Panel Data and Analysis

This study takes the panel data consisting of both dependent (response) variables (“CO_2_ emissions”, “renewable electricity output”) and the seven independent (explanatory) variables from twenty-six OECD countries from 1987 to 2013. Panel analysis has the advantage of solving the small-N problem, which is a disadvantage of comparative studies between countries [40]. There are various panel models, but this study adopts the PCSE (panel corrected standard errors) model. Generally, panel data is likely to cause problems of heteroscedasticity of error terms, autoregression, and simultaneous correlation. To solve these problems, we adopt the PSCE model proposed by Beck and Katz [41]. This model is generated via the dynamic model, using the following equation:

Yit = ρYi,t − 1 + βXi, t + εit: i = 1, …, N (t = 1, …, T)(1)

In other words, PCSE treats the problem of heteroscedasticity of the error term and the simultaneous correlation between panel groups. In addition, it is possible to calculate the standard error of panel correction considering autocorrelation. Here, PCSE shows that the estimated model fits. 

This study notes that Beck and Kartz argue that use of the dynamic model eliminates serial correlation [41]. In panel analysis, only the Hausman test is used to distinguish the random and fixed effect model. The PCSE model has been mainly used without any tests in cross-country comparative studies.

### 3.2. Measurement Framework

In this paper, the OECD countries were selected for analysis. In the OECD countries, statistical data is highly reliable and collection is relatively easy. In addition, in terms of ecological modernization, economic growth can be assumed to some extent [42]. Carbon dioxide (CO_2_) emissions accounts for most of the greenhouse gases emitted by economic growth [43]. This is because they are related to manufacturing activities and are useful for discussing ecological modernization aimed at increasing economic growth and overcoming ecological crisis. In addition, CO_2_ is more suitable for empirical analysis, with well-established data and a small error range. Recently, the OECD countries have been expanding renewable energy sources instead of fossil fuels (which produce much CO_2_) to protect the environment [44].

Renewable energy is one of the most important drivers for achieving environmentally sound and sustainable development (ESSD). In addition, many conceptual frameworks linked to sustainability are formed at economic and social levels [45]. Some existing research on renewable energy [44,46,47] has revealed that the driving force behind renewable energy is derived from the social and economic context based on the SD perspective. Therefore, “CO_2_ emissions” and “renewable electricity output” are selected as the dependent variables. Data on “CO_2_ emissions” per capita and “renewable electricity output” (% of total electricity output) were extracted from the World Bank’s World Development Indicator Database. The absolute value of CO_2_ emission is larger than that of other variables and is converted to a log value.

In terms of independent determinants, first in the economy category, “GDP per capita” is the first major variable representing each country’s economic situation. In this analysis, per capita GDP growth rate is an independent variable. Compared to the commonly-used GDP level indicator per capita, this variable can solve the unit root problem. In other words, the US GDP per capita in 1998 is likely to be in line with that of 2010.

Looking at previous studies [48,49] that analyzed factors that affected changes in CO_2_ emissions, the analysis was performed mainly on the industrial sector, energy conversion, and transport variables. Torvanger [48] analyzed the factors of change in CO_2_ emissions for manufacturing industries in nine OECD countries and found that the decrease in energy intensity was the main cause contributing to the reduction in CO_2_ intensity. According to Greening [49], analysis of ten OECD countries showed that per capita GDP and manufacturing ratios affected fuel substitution, and renewable energy was a factor in reducing CO_2_ emissions. Therefore, the number of workers in the manufacturing industry was included in the economic variable.

As mentioned in the previous cases of UNCED (UN Conference on Environment and Development) in 1992, at the Kyoto Protocol of UNFCCC (UN Framework Convention on Climate Change) in 1997, and the WSSD (World Summit of Sustainable Development) in 2002, efforts to reduce CO_2_ emission through international agreements were made. Here, it is possible to estimate the total trade (sum of import and export) as a percentage of GDP, and this was selected as the globalization variable. The total trade between the countries participating in the agreement is high. In addition, it has been shown that the amount of trade, globalization, and environmental variables are closely related [50].

Second, in relation to the social category, this paper highlights that the driving force that promotes renewable energy comes from social and economic pressures [44,46]. There is also an argument that many conceptual frameworks linked to environmental sustainability are formed on social and economic levels [45]. Therefore, this study attempts to identify the influencing factors expressed via various actors from social and economic perspectives, and examine how these factors relate to “CO_2_ emissions” and “renewable electricity output”. To this end, “social expenditure” is used as a proxy variable for social variables [51]. In the various existing studies, including those by Videras [52] and Kang et al. [53], “population” was pointed out as a major factor in CO_2_ emissions. Lastly, the left–green party variable follows the eco-socialism of Adams [36], emphasizing the green and red alliance, and was included in the analysis by estimating the interaction effect of the left and green parties of the countries subject to the OECD analysis.

A summary of the variables discussed above is shown in Table 1.

## 4. Research Findings 

### 4.1. Analytical Results 

**Figure 2:**
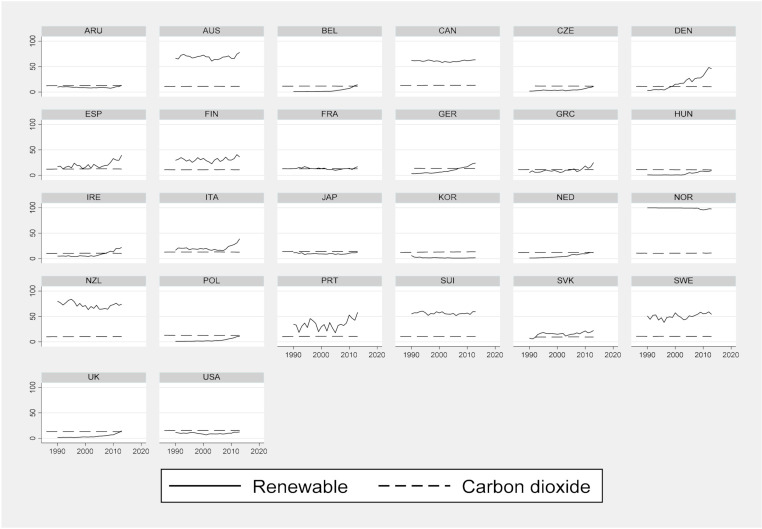
Trends by country in CO_2_ emissions and renewable electricity output (from 1987 to 2013).

Figure 2 below shows the trends in CO_2_ emissions and renewable electricity output in twenty-six OECD countries from 1987 to 2013. The amount of CO_2_ was converted to a log value, because the unit was large. The amount of fluctuation is dynamic in renewable energy compared to CO_2_ emissions. In particular, these changes are remarkable in Austria, Denmark, Finland, New Zealand, Portugal, and Sweden.

Renewable electricity output (% of total electricity output) in twenty-six OECD countries is below 50% on average, excluding New Zealand, Austria, Sweden, and Norway. Since 2010, Denmark, Sweden, Norway, and Portugal have exceeded 50%, and the overall trend is rising in most countries. CO_2_ emissions vary more across countries than within countries. In particular, the United States, Japan, China, Australia, Portugal, and the United Kingdom are located between 12% and 16%, showing a high emission trend. These countries have relatively large populations and high CO_2_ emissions in countries with advanced manufacturing industries.

Further, Nordic countries such as Finland, Denmark, and Sweden are showing a low level below 12%. This level is also at 12% or higher in Australia, while New Zealand is relatively low at 10%. The upward trend is high in Korea (South). Although there are differences between countries, both CO_2_ emissions and renewable electricity output are showing an overall upward trend over time.

### 4.2. Correlationship Analysis

**Figure 3:**
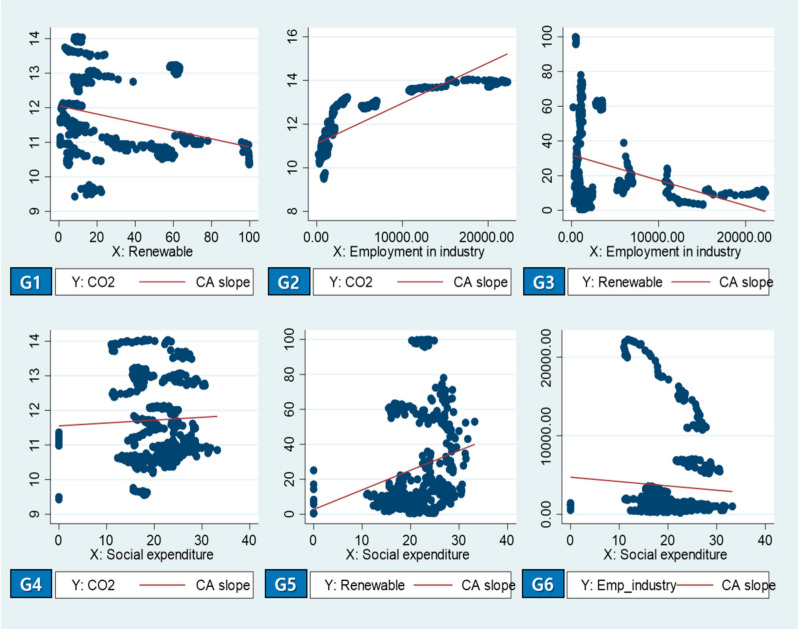
Correlation analysis results of the relevant variables (1987–2013).

As described in Figure 3 and Table 2 below, the correlation analysis (CA) showed statistically significant correlations for the economic, social, and environmental variables, while expressing insignificant correlation for the green party variable. Positive correlations were shown between “CO_2_ emissions” and “employment in industry” (see G2), and “renewable electricity output” and “social expenditure” (see G5). On the other hand, there was a negative correlation between “renewable electricity output” and “employment in industry” (see G3), and “Social expenditure” and “employment in industry” (see G6).

The correlation analysis was carried out and revealed significant results among “CO_2_ emissions”, “renewable electricity output”, “employment in industry”, “social expenditure”, and “green party” by the OECD countries. The correlation analysis looked at the correlation between two variables, and there is a difference in causality. As shown in Figure 3, the correlation analysis result was statistically significant, with the slope of -4 (see G1) on the increase in CO_2_ emission and renewable electricity output.

In particular, co-relationships were statistically significant with “CO_2_ emissions”, “employment in industry”, and social expenditure with the slopes of +0.84 (see G2) and 0.19 (see G4), respectively. However, “renewable electricity output” showed a significant relationship with “employment in industry” and “social expenditure” with the slopes of −0.29 (see G3) and +0.2 (see G5), respectively, which showed a different direction from CO_2_ emissions.

The correlation between most of the environmental variables was statistically significant, but no significant correlation was found for justifiable variables such as “Green Party”.

Table 3 below describes the results of panel analysis. The analysis was performed from 1986 to 2013 according to the previously described PCSE (panel corrected standard errors) model. In particular, the panel analysis was conducted by dividing the period into three: “1987–2013” (entire period), “1987–2002” (first period), and “2003–2013” (second period). This is based on the years (1987 and 2002) in which the historical agreements were made: the World Conference on Environment and Development (WCED, the first publicly accredited conference of SD) and the World Summit of Sustainable Development (WSSD, the first global summit of national leaders to discuss SD)—previously explained in Section 2.

First, looking at the result during the entire period (1987–2013), it did not appear that the economic and social variables had a positive impact on reducing CO_2_ emissions. This reveals that “real GDP” and “population” are the variables that influence CO_2_ emissions in a negative way. This may imply the coupled link between economic growth (GDP) and greenhouse gas emissions; namely, as economic growth increases, the emission increases. However, in relation to the renewable electricity output during the entire period, “employment in industry” and “social expenditure” are variables that had a positive impact.

During the first period (1987–2002), “employment in industry” and “social expenditure” appeared to have a positive effect on renewable electricity output. However, “employment in industry” and “population” variables negatively affect CO_2_ emission; in other words, as they increase, CO_2_ emissions increase. Moreover, “globalization” and “population” variables negatively affect renewable electricity output. 

In particular, the analysis of the second period (2003–2013) reveals that “globalization” and “social expenditure” are positive factors influencing the reduction of CO_2_ emission, and “employment in industry” has a positive effect on renewable electricity output. However, four variables during the second period had negative effects on both the reduction of CO_2_ reduction and the increase of renewable electricity output, as follows: “real GDP”, “employment in industry”, and “population” negative for CO_2_ emission reduction; “globalization” and “population” negative for renewable electricity output increase. 

Therefore, the findings are different for each set period. “Globalization” and “social Expenditure” appeared to have a positive effect on the reduction of CO_2_ emissions in the second period (2003–2013), unlike the results for the entire period (1987–2002). Meanwhile, the following three variables gave the same results in the all three periods (1987–2013, 1987–2002, and 2003–2013); “employment in industry” positively affects renewable electricity output; “mployment in industry” and “population” have a negative impact on the reduction of CO_2_ emissions; and “globalization” and “population” negatively affect renewable electricity output. Therefore, the population variable was found to have a negative impact on the both response variables (CO_2_ emissions and renewable electricity output), regardless of the duration.

## 5. Conclusions and Implications

This study has attempted to analyze how the three pillars of sustainable development (SD; economic growth, social justice(welfare), and environmental protection) have influenced each other for the past twenty-six years (from 1987 to 2013). The relationship between the triangular pillars of SD, which has been selected as the Sustainable Development Goals (SDGs) to be achieved by United Nations member states by 2030, can be particularly characterized by “ecological modernization” and “eco-socialism”. This study differs from the existing studies in that it explains multiple dimensions of SD by including not only the theory of ecological modernization but of ecological socialism. Furthermore, this paper empirically proved the theoretical multifaceted dimensions through the panel data analysis.

This paper examined the correlation analysis of the nine representative variables in three categories, introducing the cases of twenty-six OECD countries. In particular, the panel analysis (PCSE models) was conducted to identify the independent (explanatory) determinants affecting the dependent (response) variables, i.e., the environmental factors (CO_2_ emissions and renewable electricity output). As a result, during the entire period (1987–2013) the findings reveal that the all economic and social variables did not have a positive impact on reducing CO_2_ emissions. However, “employment in industry” and “social expenditure” are variables that affected the increase of renewable electricity output. Additionally, the population variable had a negative impact on the both response variables.

In particular, this study notes that the findings’ results appeared differently for each set period (1987–2013, 1987–2002, and 2003–2013). It tries to suggest the following implications in relation to the detailed findings, depending on the three set periods. First, in light of ecological modernization (the side AB of the triangle of SD in Figure 1 above), this paper highlights that there is a coupled relationship of economic growth (the variable of Real GDP) and CO_2_ emissions during the entire period (1987–2013). This study, in short, has implications that are in line with the results revealed in Huh’s research [58]; the inverse relationship between sustained economic growth and greenhouse gas (including CO_2_ emissions reductions). Therefore, it is contrary to the context of ecological modernization.

Second, the result of the relationship between the variable of employment of industry and the renewable electricity output follows the context of ecological modernization. The consequence of the positive influence highlights that the expansion of the manufacturing industry, symbolized by the increase in the number of employees employed, is connected with the development of the renewable energy industry. In other words, it can be seen that the renewable energy industry including solar power and wind power is vastly affected by the manufacturing industry [9,59]. For example, in order to supply renewable energy such as solar and wind power, solar cells, modules, and wind power generators should be produced, which is closely related to the chemical product industry and primary metal product industry sectors of the manufacturing industry [59,60]. Meanwhile, the result between employment of industry and CO_2_ emissions (the positive relationship between the two variables) can be regarded as the same as those of previous studies [61,62].

Third, this study notes that during the second period (2003–2013), the increase in globalization (openness of the economy and international trade) had a positive effect on the reduction of CO_2_ emissions. This result might be seen as a positive reflection of various international efforts during the period after the Kyoto Protocol of UNFCCC (UN Framework Convention on Climate Change) in 1997, the WSSD (World Summit of Sustainable Development) in 2002, and the Rio+20 UN Conference in 2012.

The international community’s efforts to reduce greenhouse gas (including CO_2_) emissions have been linked to efforts to expand trade in environmental products [63]. For example, in the 2010s, 14 member states of the World Trade Organization (WTO) (including the United States, EU, Canada, Korea, and Japan) conducted EGA (Environmental Goods Agreement) negotiations to lower tariffs and other trade barriers on environmental products [63,64]. In particular, the United States launched the Major Economies Forum on Energy and Climate in 2009 and promoted free trade in environmental products and services with 17 major countries that account for 75% of greenhouse gas emissions [64].

Meanwhile, the measures such as technical regulations and standards, border coordination measures, and the flexibility systems (including the Joint Implementation, the Clean Development Mechanism, and the Emission Trading) introduced by GHG (greenhouse gas) obligation reduction countries have also had a great influence on trade and investment policies [65,66]. Since the major developed economies of the OECD are both carbon emitters and parties to the UNFCCC, efforts have been pursued in a variety of ways to successfully establish environmental and trade policy links.

**Figure 4:**
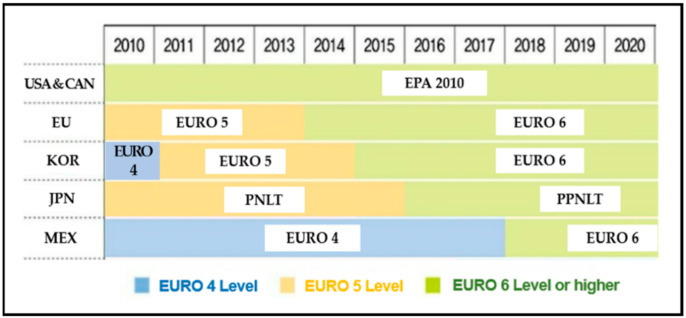
Level of environmental regulations for commercial vehicles in major oecd countries. Sources: Korea Economic Research Institute, 2017 [29].

For example, the reduction of CO_2_ emissions from automobiles has been attracting worldwide attention based on the 1997 Kyoto Protocol, and has been implemented as a key element of GHG reduction strategies, especially in EU countries [67]. The annual transport sector of CO_2_ emissions by OECD countries increased overall by 2004 (e.g., 13-year (1990–2003) growth rate: Korea 98.2%, Canada 28.6%, and the United States 18.3%) [60]. However, since 2005, it has been shown to continue to decline in the OECD countries (especially the US and EU countries). This may be seen as the effect of the reinforcement of the global automobile emission regulation norms [68,69,70]. In particular, around 2010, the strengthening of automobile emission regulations in major countries affected automobile imports and exports [29,61]. Namely, the increase in global automobile trade since 2010 may have an impact on the reduction of CO_2_ emissions. As shown in Figure 4 below, major OECD countries raised the level of emission regulation around 2010. 

For example, the United States and Canada have applied the most stringent emission-related regulations in the world [29,59]. In 2010, the US Environmental Protection Agency (EPA) enacted its own emission regulation “EPA2010” (‘Euro 6” level). This regulation contains more stringent regulations than Euro 6 (applied from 2014 in the EU; NOx emission less than 0.4 g/kWh, CO_2_ emission less than 1.5 g/kWh, PM emission less than 0.01 g/kWh). In addition, from 2005 major European countries have also applied the “Euro 4” stage (NOx emission less than 3.5 g/kWh, CO_2_ emission less than 1.5 g/kWh, PM emission less than 0.02 g/kWh). From 2008, the “Euro 5” stage (NOx emission less than 2.0 g/kWh, CO_2_ emission less than 1.5 g/kWh, PM emission less than 0.02 g/kWh) began to be applied. Currently, twenty-seven countries in the European Union are applying “Euro 6” regulations (adopted in 2014). Mexico has applied “Euro 4” since mid-2000, and introduced “Euro 6” from 2018. In the case of Japan, the PNLT, own emission regulation, as same as the level of Euro 5, has been adopted since 2009, and in 2016, the PPNLT regulation, at the same level as Euro 6, was introduced. In addition, Korea followed the Euro 4 regulation until 2010, the Euro 5 regulation until 2015, and has been leading the way in Northeast Asia by introducing the Euro 6 since 2016 [29,59].

In terms of eco-socialism (the side BC of the triangle of SD, in Figure 1 above), this paper underlines that during the entire period 1987–2013), “social expenditure” positively affected the increase of renewable electricity output, and during the first period (1987–2002), the variable of social expenditure appear to have a positive effect on the decease of CO_2_. Since a positive causal relationship between environmental protection and social welfare (justice) has been revealed, the research results may have significant implications for environmental welfare policy.

Environmental welfare can be defined as guaranteeing the right to access to the minimum environmental quality and services for human life for all citizens, and ensuring environmental stability and providing a pleasant environment [65,66]. Through the promotion of environmental welfare policies, it is possible to diversify policies that focus on the environmentally-vulnerable people, such as investing in social overhead capital (SOC) and improving social services to reduce these risks. Ultimately, priorities can be adjusted by reviewing the overall environmental policies [71,72]. In addition, apart from the traditional, post-health policy focused on the individual, policies can be expanded to a healthcare policy that integrates the regional and local environment.

However, although there are significant findings, this study has limited ability to explore all cases of the thirty-seven OECD countries and only analyzed only twenty-six countries (approximately 70% of the total) capable of collecting relevant data. In addition, “green party’ and “left party” variables were selected as the independent variables in order to understand the consequences of the integrated effect of the economic and social sectors on the dependent (result) variable (environmental factor), but there are limitations in which no meaningful results were revealed. To overcome these limitations, various follow-up studies will be needed to study the relevant contexts of each OECD country’s detailed case in relation to the three pillars (economic growth, social justice, and environmental protection) of sustainable development.

## Figures and Tables

**Table 1:** The Variable Framework.

	Categories		Operationalization	Source
Dependent Variables	Environment	CO_2_ emissions	CO_2_ emissions (metric tons per capita) 1987~2014	World bank dataset (2020) [54]
		Renewable electricity output	Renewable electricity output (% of total electricity output) 1987~2014	World bank dataset (2020) [55]
Independent Variables	Economy	Real GDP	Growth of real GDP, percentage change from previous year	Armingeon et al. (2019) [56]
		Employment in industry	Civilian employment in industry, in thousands	Armingeon et al. (2019) [56]
		Globalization	Openness of the economy, measured as total trade (sum of import and export) as a percentage of GDP at current prices	Armingeon et al. (2019) [56]
	Society	Social expenditure	All social expenditure per capita	OECD (2020) [57]
		Population	Total population, in thousands.	Armingeon et al. (2019) [56]
		Left party	Government support: parliamentary seat share of social democratic and other left-leaning parties in government	Armingeon et al. (2019) [56]
		Green party	Government support: parliamentary seat share of green parties in government.	Armingeon et al. (2019) [56]

**Table 2:** Correlation analysis results of the relevant variables (1987–2013).

	CO_2_				Renewable Electricity Output			Employment in Industry
	Renewable Electricity Output	Employment in Industry	Social Expenditure	Green Party	Employment in Industry	Social Expenditure	Green Party	Social Expenditure
correlation coefficient	−0.4055 *** (0.001)	0.8412 *** (0.0000)	−0.2111 *** (0.000)	−0.071 (0.06)	−0.2905 *** (0.0000)	0.2027 *** (0.0000)	0.1047 (0.010)	−0.2111 *** (0.0000)

*p* value < 0.01—***.

**Table 3:** Determinants of CO_2_ emissions and renewable electricity output.

Dependent Variables		CO_2_ Emissions (log Value)			Renewable Electricity Output		
		PCSE 1987–2013	PCSE (1st)87–2002	PCSE (2nd)03–13	PCSE 1987–2013	PCSE (1st)87–2002	PCSE (2nd)03–13
Economy	Real GDP	0.005 ***	0.001	0.006 ***	0.156 *	0.236	0.306 *
	Globalization	−0.001	0.000	−0.002 ***	−0.101 ***	−0.226 ***	−0.186 ***
	Employment in industry	0.002 ***	0.002 ***	0.01 ***	0.0001 *	0.002 ***	0.002 ***
Society	Social expenditure	0.001	0.002	−0.017 ***	0.264 **	0.353 ***	0.575
	Population	0.995 ***	0.986 ***	0.992 ***	−13.378 ***	−17.473 ***	−16.175 ***
	Green * Left party	−0.000	−0.000	0.000	0.001	0.003	−0.001
	_cons	2.453 ***	2.507 ***	3.068 ***	15.062 ***	191.464 ***	177.630 ***

*p* value < 0.01—***; 0.05—**; 0.1—*.

## Data Availability

All data source in Table 1 (refer to reference [54,55,56,57]).
